# Probable Primary Adrenal Insufficiency Associated With Granulomatous Adrenal Calcifications, Non-tuberculous Mycobacterial Ileitis, and Crohn’s Disease: A Case Report

**DOI:** 10.7759/cureus.111577

**Published:** 2026-06-26

**Authors:** Gilberto M Lozano Dubernard, Perla J Ruíz López, Francisco I Aguiar Hernández, Javier López Gutiérrez

**Affiliations:** 1 General Surgery, Hospital Ángeles Pedregal, Mexico City, MEX

**Keywords:** adrenal gland, crohn's disease, extrapulmonary, granuloma, ileum, non-tuberculous mycobacterium, primary adrenal insufficiency, tuberculosis

## Abstract

Granulomatous adrenal involvement is an uncommon cause of primary adrenal dysfunction, while intestinal non-tuberculous mycobacterial (NTM) infection and Crohn's disease share substantial clinical, endoscopic, radiological, and histopathological overlap. The coexistence of these entities represents an exceptional diagnostic challenge, and epidemiological data regarding their association are currently lacking.

We report the case of a 61-year-old man who presented with progressive cutaneous hyperpigmentation. Computed tomography demonstrated bilateral adrenal calcifications, calcified lymph nodes suggestive of granulomatous disease, and a calcified hepatic granuloma. Biochemical evaluation revealed markedly elevated adrenocorticotropic hormone (ACTH) levels, while morning cortisol and aldosterone

## Introduction

Addison’s disease is primary adrenal dysfunction and is characterized by a marked decrease in cortisol levels accompanied by an increase in adrenocorticotropic hormone (ACTH) levels. Hyponatremia, hypoglycemia, and hypotension are often observed in subjects with Addison’s disease. Pigmentation of the face and hands may also occur and is thought to result from increased ACTH and/or melanocyte-stimulating hormone secretion through the cortisol deficiency-mediated feedback system. Although autoimmune adrenal destruction represents the most common etiology, infectious diseases remain an important cause of primary adrenal insufficiency worldwide [1].

Approximately 75%-80% of cases are caused by autoimmune destruction of the adrenal gland, whereas tuberculosis accounts for 7%-20% of cases of Addison’s disease. Among infectious causes, tuberculosis has historically been recognized as one of the leading etiologies of adrenal insufficiency, particularly in regions where mycobacterial infections remain prevalent [2]. When more than 90% of the adrenal cortex is destroyed, Addison’s syndrome appears. Tuberculosis more commonly involves both adrenal glands than a single gland. The presence of adrenal atrophy concomitant with calcification has sometimes been observed in patients with long-standing tuberculosis, and the incidence of calcification increases with disease duration. A possible explanation is that, during the late stages of tuberculosis, encapsulated granulomas become quiescent, inflammatory cell infiltration decreases, and calcium salts are deposited within caseous material [3].

While *Mycobacterium tuberculosis* is a well-established cause of adrenal involvement, non-tuberculous mycobacteria (NTM) represent a distinct group of environmental organisms that have increasingly emerged as human pathogens. NTM infection primarily manifests as pulmonary disease, although it can affect virtually any organ system. Previous data indicate that 20%-30% of NTM infections originate from extrapulmonary sites. Extrapulmonary NTM disease often affects a younger population but can occur at any age, with a similar distribution between males and females [4].

Despite the increasing recognition of extrapulmonary NTM disease, adrenal involvement remains exceedingly uncommon. A review of the available literature did not identify any studies reporting the incidence or prevalence of Addison’s disease associated with non-tuberculous mycobacterial infection. Existing evidence is restricted to sporadic case reports, which precludes the calculation of meaningful epidemiological estimates. Accordingly, the true frequency of this association remains unknown [1].

Beyond adrenal disease, the potential role of mycobacterial organisms in chronic inflammatory conditions has been the subject of ongoing investigation. Crohn’s disease (CD) is one such condition in which an infectious contribution has been proposed but remains controversial. Crohn’s disease is a chronic inflammatory bowel disease that can affect any segment of the gastrointestinal tract but is typified by terminal ileal involvement, transmural inflammation, and non-necrotizing granulomas. Despite certain pathological similarities to mycobacterial diseases, acid-fast bacilli are rarely identified in Crohn’s disease [5].

The relationship between Crohn’s disease and infection remains complex and incompletely understood. The disease may develop as a reaction to a persistent intestinal infection, a defective mucosal barrier to luminal bacterial or non-bacterial antigens, or a dysregulated host immune response to ubiquitous pathogens. It remains unclear whether the disease is driven by a specific microorganism or by a broader disturbance of the intestinal microbiota [6].

Consequently, differentiating intestinal mycobacterial infection from Crohn’s disease remains a major diagnostic challenge because of overlapping clinical, radiological, endoscopic, and histopathological features. The coexistence of primary adrenal insufficiency, granulomatous adrenal calcifications, non-tuberculous mycobacterial ileitis, and Crohn’s disease is exceptionally uncommon, and epidemiological data regarding this association are currently unavailable. We present the case of a patient with primary adrenal insufficiency associated with bilateral adrenal calcifications, ileal stenosis caused by non-tuberculous mycobacterial infection, and coexisting Crohn’s disease.

## Case presentation

A 61-year-old male presented to the clinic with a six-month history of progressive hyperpigmentation affecting the face, flexural areas, left abdominal flank, and lumbar region. His medical history was notable for daily consumption of unpasteurized milk during childhood and a previous left hemicolectomy due to complicated diverticular disease at 52 years old. Additional history revealed no tobacco use, alcohol consumption, or prior exposure to immunosuppressive therapy or history of fever. He denied constitutional symptoms commonly associated with adrenal insufficiency, such as fatigue and unintentional weight loss. On presentation, the patient’s vital signs were within normal limits, without evidence of hypotension. Physical examination did not demonstrate peripheral lymphadenopathy. Laboratory results obtained during hospital admission are shown in Table 1.

**Table 1:** Laboratory findings at admission Baseline laboratory findings obtained during the initial diagnostic evaluation. Abnormal values are indicated by arrows (↑ elevated, ↓ decreased). TSH: Thyroid-Stimulating Hormone; BUN: Blood Urea Nitrogen; ACTH: Adrenocorticotropic Hormone; PPD: Purified Protein Derivative; AST: Aspartate Aminotransferase; ALT: Alanine Aminotransferase; ALP: Alkaline Phosphatase; LDH: Lactate Dehydrogenase; GGT: Gamma-Glutamyl Transferase.

Category	Laboratory Test	Patient Value	Reference Range
Hematology	Leukocytes (×10³/µL)	8.78	4.0-10.0
Hematology	Neutrophils (%)	56	40-70
Hematology	Lymphocytes (%)	31.7	20-40
Hematology	Hemoglobin (g/dL)	18.09 ↑	13.5-17.5
Hematology	Hematocrit (%)	54.4 ↑	41-53
Biochemistry	Creatinine (mg/dL)	2.1 ↑	0.7-1.3
Biochemistry	BUN (mg/dL)	19	8.4-25.7
Biochemistry	Urea (mg/dL)	40.7	10.7-53.5
Biochemistry	Glucose (mg/dL)	107 ↑	70-99
Biochemistry	Uric acid (mg/dL)	9.3 ↑	3.5-7.2
Biochemistry	Triglycerides (mg/dL)	289 ↑	<150
Biochemistry	Calcium (mg/dL)	10.2	8.5-10.5
Endocrine Profile	T3 (ng/dL)	109.1	80-200
Endocrine Profile	T4 (µg/dL)	7.2	5.0-12.0
Endocrine Profile	Free T4 (ng/dL)	0.88	0.8-1.8
Endocrine Profile	TSH (µIU/mL)	5.11 ↑	0.4-4.5
Endocrine Profile	Morning cortisol (ng/mL)	95	50-250
Endocrine Profile	ACTH at presentation (pg/mL)	952 ↑	7.2-63.3
Endocrine Profile	ACTH during follow-up, 2025 (pg/mL)	234.9 ↑	7.2-63.3
Endocrine Profile	ACTH during follow-up, 2026 (pg/mL)	24	7.2-63.3
Endocrine Profile	Serum aldosterone (pg/mL)	50.1	30-160 (supine); 70-300 (standing)
Inflammatory Markers	Fecal calprotectin (µg/g)	571 ↑	<50
Immunological Assessment	PPD at 24 hours (mm)	40 ↑	≤10
Immunological Assessment	PPD at 48 hours (mm)	30 ↑	≤10
Immunological Assessment	PPD at 72 hours (mm)	30 ↑	≤10
Catecholamines	Norepinephrine (pg/mL)	617	70-750
Catecholamines	Epinephrine (pg/mL)	<15	<110
Catecholamines	Dopamine (pg/mL)	<30	<30
Electrolytes	Sodium (mEq/L)	141	135-145
Electrolytes	Potassium (mEq/L)	4.40	3.5-5.0
Electrolytes	Chloride (mEq/L)	106	98-107
Liver Function	Total bilirubin (mg/dL)	1.0	0.2-1.2
Liver Function	Direct bilirubin (mg/dL)	0.3	0.0-0.3
Liver Function	Indirect bilirubin (mg/dL)	0.7	0.2-0.9
Liver Function	AST (U/L)	30	10-40
Liver Function	ALT (U/L)	47	7-56
Liver Function	ALP (U/L)	53.1	44-147
Liver Function	LDH (U/L)	101 ↓	140-280
Liver Function	GGT (U/L)	29	9-48
Serology	Anti-HIV antibody	Non-reactive	Non-reactive

**Figure 1:**
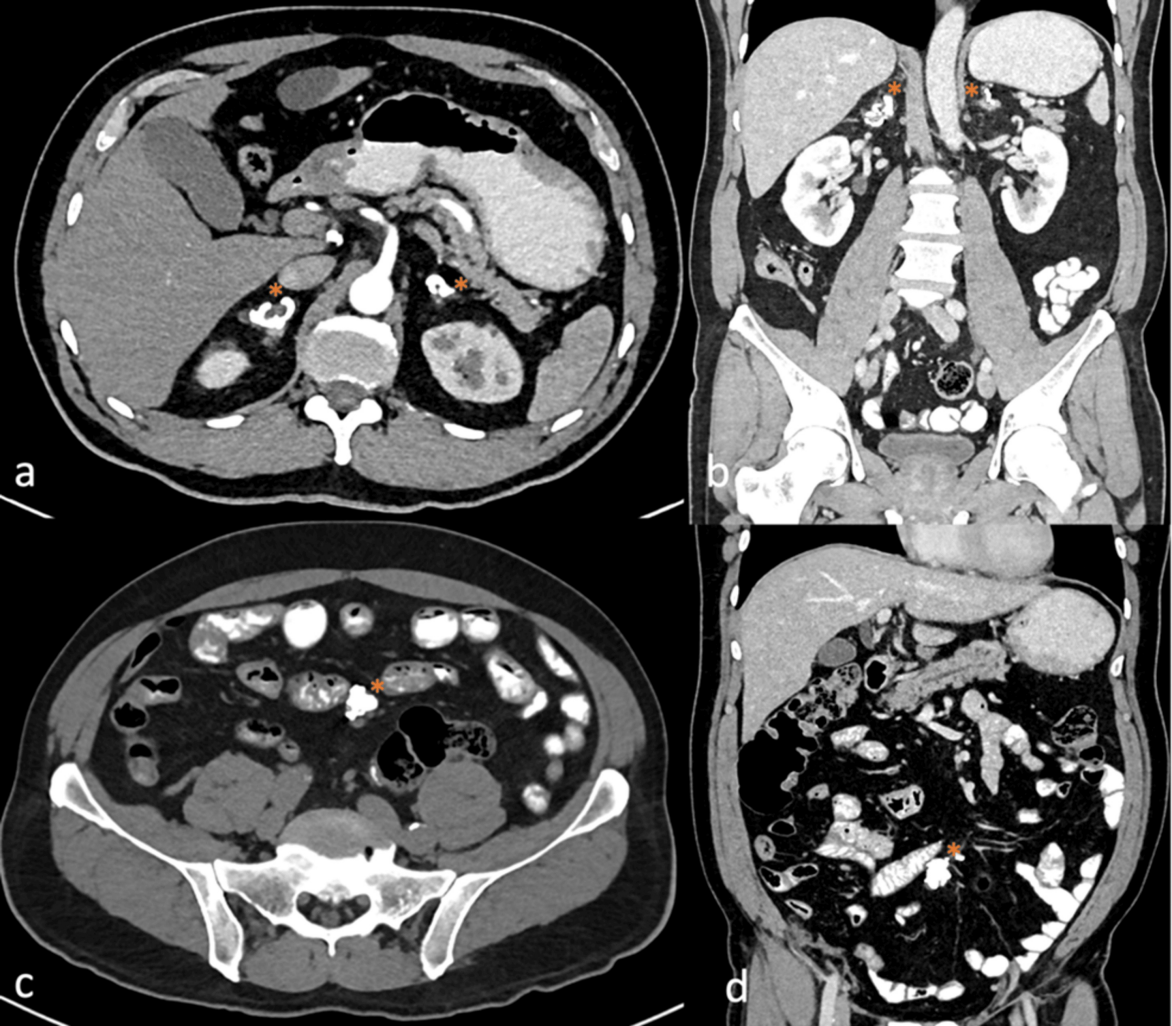
Adrenal and mesenteric calcifications a. Bilateral adrenal calcifications (* indicates bilateral adrenal calcifications). b. Ectopic adrenal tissue adjacent to the upper pole of the left kidney (* indicates bilateral adrenal calcifications). c. Axial view of mesenteric calcification (* indicates mesenteric calcification suggestive of granulomatous disease). d. Mesenteric calcification suggestive of granulomatous disease (* indicates mesenteric calcification suggestive of granulomatous disease).

Abdominal computed tomography (CT) revealed bilateral adrenal calcifications and calcified lymph nodes suggestive of granulomatous disease. An ectopic adrenal tissue adjacent to the upper pole of the left kidney was also identified (Figure 1). Additionally, a calcified hepatic granuloma was observed in segment VII.

Given the patient's marked cutaneous hyperpigmentation, markedly elevated ACTH levels, and the radiologic findings of bilateral adrenal calcifications associated with calcified lymph nodes suggestive of granulomatous disease, granulomatous adrenal involvement leading to probable primary adrenal insufficiency was considered. Because mycobacterial infection is a recognized cause of granulomatous adrenal disease, further evaluation for tuberculosis was undertaken, including purified protein derivative (PPD) testing. PPD testing showed induration measuring 40 mm at 24 hours, 30 mm at 48 hours, and 30 mm at 72 hours.

The initial diagnostic impression was established through the integration of clinical, biochemical, and radiological findings. Although morning serum cortisol and aldosterone levels were not unequivocally diagnostic and dynamic endocrine testing was not performed, the constellation of marked hyperpigmentation, profound ACTH elevation, bilateral adrenal calcifications, and a hyperergic PPD reaction strongly suggested probable primary adrenal insufficiency associated with granulomatous disease. Based on these findings, and considering the potential consequences of untreated adrenal dysfunction, corticosteroid therapy was initiated. The patient was first treated with hydrocortisone 10 mg in the morning and 5 mg in the evening (total 15 mg daily), subsequently switched to deflazacort 6 mg once daily.

**Figure 2:**
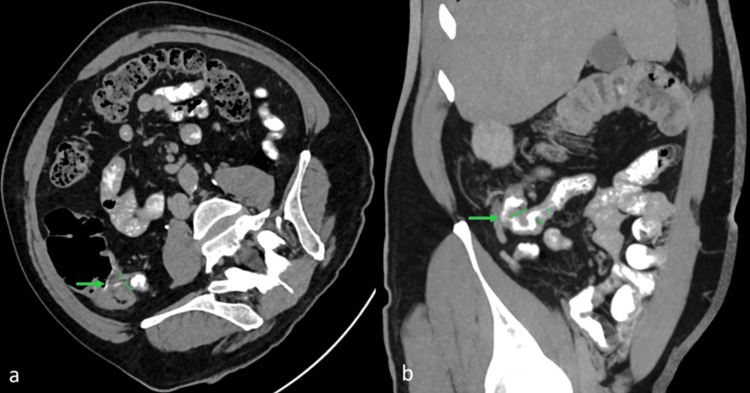
Tomographic findings in the terminal ileum region a. Ileal stenosis (arrow indicates the site of ileal stenosis). b. Asymmetric and irregular wall thickening, consistent with nonspecific terminal ileitis of undetermined etiology (arrow highlights asymmetric and irregular thickening of the terminal ileal wall, with loss of normal mural architecture, consistent with nonspecific terminal ileitis of undetermined etiology).

Four months later, the patient returned to the clinic reporting progressive abdominal distension, intermittent diarrhea, and pain localized to the right flank and right iliac fossa. Abdominal ultrasound demonstrated thickening of the ileal wall measuring 8 mm at the ileocecal valve, with decreased motility. A contrast-enhanced CT scan revealed ileal stenosis associated with asymmetric and irregular wall thickening, along with loss of normal mucosal architecture, consistent with nonspecific terminal ileitis of undetermined etiology (Figure 2).

**Figure 3:**
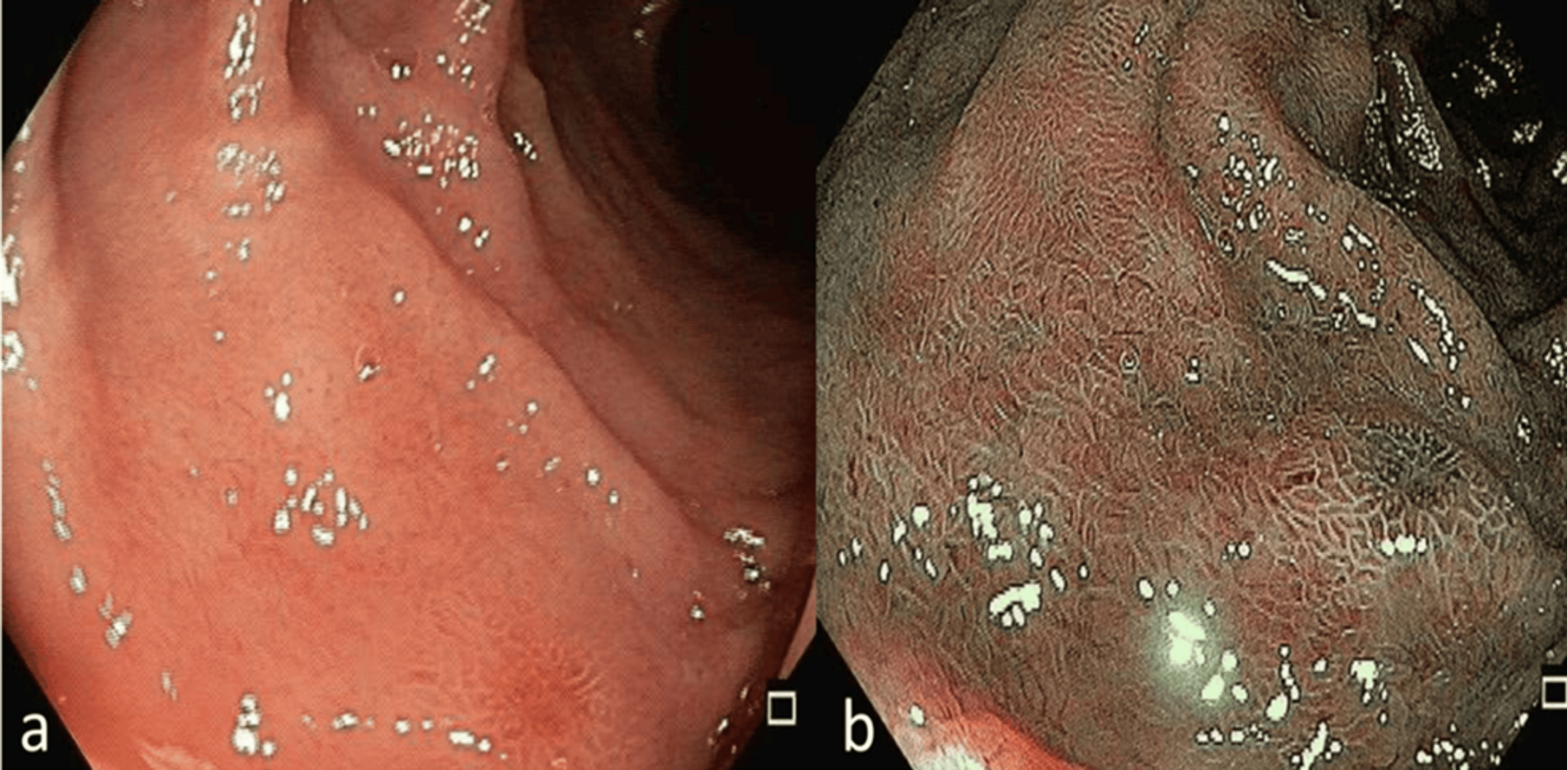
Endoscopic findings of the terminal ileum a. Endoscopic view of the terminal ileum showing ulcerations and erythema. b. Magnified endoscopic view of the same region using chromoendoscopy, highlighting mucosa details.

Subsequent ileocolonoscopy demonstrated granular mucosa in the terminal ileum with multiple linear ulcers, further supporting the presence of active inflammatory involvement of the ileocecal region (Figure 3).

Histopathological analysis of ileal biopsy specimens obtained during Ileocolonoscopy revealed deep, active, and ulcerated chronic ileitis. Real-time PCR performed on the gastrointestinal tissue following genomic DNA extraction detected non-tuberculous Mycobacterium. The assay utilized fluorescent-labeled primers, positive and negative controls, and amplification of an internal reaction control to ensure assay validity. However, species-level identification of the non-tuberculous mycobacteria was not available. Antineutrophil cytoplasmic antibodies (ANCA) and anti-saccharomyces cerevisiae antibodies (ASCA) were negative. Quantitative fecal calprotectin level was 571 µg/g. HIV enzyme-linked immunosorbent assay (ELISA) was nonreactive.

Given the molecular detection of mycobacterial DNA in ileal tissue, together with the patient's history of granulomatous adrenal involvement, bilateral adrenal calcifications, and a markedly hyperergic PPD reaction, anti-tuberculosis therapy was initiated using a fixed-dose combination tablet containing rifampicin (150 mg), pyrazinamide (400 mg), ethambutol hydrochloride (300 mg), and isoniazid (75 mg). A nine-month treatment was planned, and its completion was subsequently confirmed during follow-up. Following a multidisciplinary evaluation, the patient underwent a laparoscopic right hemicolectomy for ileal stenosis with asymmetric and irregular mural thickening affecting the distal 15 cm of the terminal ileum, followed by a side-to-side ileocolic anastomosis to the transverse colon. During the procedure, multiple granulomatous lesions were observed in the mesentery of the terminal ileum and at the root of the mesentery.

The surgical specimen was sent for histopathological examination, which macroscopically revealed a 3-cm diameter ulcerated lesion with thickened borders in the ileum, located 2 cm from the ileocecal valve. Microscopically, glandular distortion, granulomas, pyloric-type metaplasia without dysplasia, and 15 lymph nodes without abnormalities were observed. After surgery, the patient had a favorable postoperative course. The subocclusive symptoms resolved completely. The patient continues under multidisciplinary outpatient follow-up and is still receiving treatment with deflazacort 6 mg once daily. At the last follow-up, ACTH levels had normalized, decreasing from 952 pg/mL at baseline to 24 pg/mL, while the patient continues deflazacort treatment, accompanied by sustained clinical improvement.

## Discussion

This case illustrates the diagnostic challenges posed by the coexistence of intestinal non-tuberculous mycobacterial (NTM) infection, Crohn's disease, and granulomatous adrenal involvement. The overlap of these entities, coupled with their nonspecific manifestations, can delay recognition and complicate clinical decision-making, particularly when the presentation does not fulfill the classical diagnostic criteria of primary adrenal insufficiency.

Clinical presentation of primary adrenal insufficiency is often nonspecific and insidious, frequently resulting in substantial diagnostic delays. Unlike the classical descriptions reported in the literature, our patient did not exhibit the full spectrum of characteristic manifestations. Instead, the clinical picture was characterized primarily by marked cutaneous hyperpigmentation, elevated ACTH levels, and radiological findings of granulomatous adrenal involvement, including lymph nodes, that prompted further investigation for an underlying granulomatous process affecting the adrenal glands.

Although weight loss, gastrointestinal symptoms, hypotension, electrolyte abnormalities, and hyperpigmentation have traditionally been regarded as hallmark features of primary adrenal insufficiency, their absence does not exclude the possibility of an adrenal dysfunction. Despite the availability of advanced molecular and imaging techniques, a detailed medical history and the integration of biochemical and radiological findings remain essential components of the diagnostic approach. In our patient, this comprehensive assessment was crucial in raising suspicion of probable primary adrenal insufficiency associated with granulomatous disease, despite the absence of definitive endocrine confirmation through dynamic testing. This case further highlights that granulomatous adrenal involvement may initially manifest with incomplete clinical and biochemical features, requiring clinicians to maintain a high index of suspicion and integrate subtle clinical clues with imaging findings to avoid delays in diagnosis and treatment [7,8].

Granulomatous infections involving the adrenal glands, particularly tuberculosis, may lead to granulomatous inflammation, caseous necrosis, calcifications, and progressive destruction of the adrenal cortex. It has been reported that more than 90% of adrenal tissues must be compromised before overt clinical manifestations of adrenal insufficiency become apparent, which may explain why patients can initially present with incomplete clinical and biochemical features despite significant structural abnormalities. In most cases, adrenal involvement is bilateral, resulting from hematogenous or lymphatic dissemination from a primary infectious focus. This pattern is consistent with the bilateral adrenal calcifications and associated granulomatous lymphadenopathy observed in our patient and in previously published series [9,10].

Radiological findings represent a valuable component of the diagnostic evaluation. Computed tomography and magnetic resonance imaging may identify features suggestive of granulomatous adrenal disease, including bilateral adrenal enlargement, peripheral enhancement, central necrosis, and calcifications. Enlarged adrenal glands are more frequently associated with recent active disease, whereas atrophic or calcified glands generally suggest chronic or inactive involvement [9]. In our patient, the presence of bilateral adrenal calcifications, calcified lymph nodes suggestive of granulomatous disease, and a calcified hepatic granuloma constituted important findings that supported the suspicion of granulomatous adrenal involvement and prompted further investigation for an underlying mycobacterial etiology.

Our case also presents a particularly unusual association involving ileal disease, ileocecal stenosis, mesenteric granulomatous lesions, molecular detection of non-tuberculous mycobacteria, and histopathological findings compatible with Crohn's disease. This constellation represents a major diagnostic challenge because inflammatory bowel disease and intestinal mycobacterial infections share substantial clinical, endoscopic, radiological, and histopathological overlap.

Linear ulcers, chronic ileitis, intestinal stenosis, and granulomatous inflammation have been described in both intestinal mycobacterial infections and Crohn's disease, making it difficult to establish a definitive diagnosis based on a single isolated finding. In such scenarios, integration of clinical, microbiological, histopathological, radiological, and endoscopic data is essential to guide management and avoid potentially serious therapeutic errors, particularly when immunosuppressive therapy and antimicrobial treatment may have opposing implications [11,12].

Extrapulmonary mycobacterial infections are frequently paucibacillary, limiting the sensitivity of conventional microbiological techniques. In our patient, real-time polymerase chain reaction (RT-PCR) detected non-tuberculous mycobacterial DNA; however, species-level identification was not available, and no evidence of *Mycobacterium tuberculosis* was obtained through tissue smears, cultures, or molecular assays. Therefore, the diagnosis relied on the careful integration of clinical, radiological, histopathological, and microbiological findings rather than on a single diagnostic test.

From a therapeutic standpoint, management required a multidisciplinary approach. The coexistence of granulomatous adrenal dysfunction and suspected mycobacterial disease necessitated simultaneous hormonal replacement and antimycobacterial therapy. The patient was started on empirical anti-tuberculosis treatment using a fixed-dose combination tablet containing rifampicin (150 mg), pyrazinamide (400 mg), ethambutol hydrochloride (300 mg), and isoniazid (75 mg), with a planned treatment duration of nine months. Treatment completion was confirmed during clinical follow-up. Rifampin is known to induce the CYP3A4 enzymatic system, accelerating glucocorticoid metabolism and potentially precipitating adrenal decompensation if steroid doses are not appropriately adjusted. Therefore, recognition of these pharmacological interactions represents an essential clinical consideration during the management of this patient [7].

The persistence of subocclusive symptoms despite medical management ultimately required right hemicolectomy. Histopathological examination of the surgical specimen subsequently demonstrated inflammatory bowel disease consistent with Crohn's disease. The coexistence of NTM detection in endoscopic ileal biopsies and Crohn's disease identified in the resection specimen further underscores the complexity of differentiating intestinal mycobacterial infection from inflammatory bowel disease. Rather than establishing a definitive causal relationship between these entities, this evolving clinical course highlights the importance of continuous reassessment and integration of microbiological, pathological, radiological, and clinical findings throughout the diagnostic process. The favorable postoperative outcome and sustained clinical stability observed during follow-up support the appropriateness of the multidisciplinary approach adopted in this patient [13]. Finally, this case highlights the diagnostic complexity posed by the coexistence of probable granulomatous adrenal dysfunction, intestinal NTM infection, and Crohn's disease. Although a definitive causal relationship among these entities could not be established, maintaining a high index of clinical suspicion and systematically integrating clinical, radiological, microbiological, endoscopic, and histopathological evidence may reduce diagnostic delays and prevent inappropriate therapeutic decisions in patients with atypical presentations.

## Conclusions

This case reaffirms the value of structured clinical reasoning. The presence of granulomas should be considered a highly relevant finding; once identified, mycobacterial infection should be strongly suspected. In such cases, establishing an accurate diagnosis requires the integration of clinical, radiological, microbiological, and histopathological findings. The coexistence of non-tuberculous mycobacterial (NTM) infection, systemic granulomatosis, and histologic findings compatible with Crohn’s disease represents a complex and unusual diagnostic scenario. Differential diagnosis should include intestinal mycobacterial infection mimicking Crohn’s disease, Crohn’s disease coexisting with NTM infection, or a Crohn-like inflammatory response associated with systemic mycobacteriosis. These factors make this case particularly rare.
